# Global Pharmacovigilance for Antiretroviral Drugs: Overcoming Contrasting Priorities

**DOI:** 10.1371/journal.pmed.1001054

**Published:** 2011-07-05

**Authors:** Nyasha Bakare, Ivor Ralph Edwards, Andy Stergachis, Shanthi Pal, Charles B. Holmes, Marie Lindquist, Chris Duncombe, Alex Dodoo, Joel Novendstern, Jude Nwokike, Ricardo Kuchenbecker, Judith A. Aberg, Veronica Miller, Jur Strobos

**Affiliations:** 1 Forum for Collaborative HIV Research and Johns Hopkins Bloomberg School of Public Health, Baltimore, Maryland, United States of America; 2 WHO Collaborating Centre for International Drug Monitoring, Uppsala, Sweden; 3 School of Public Health, University of Washington, Seattle, Washington, United States of America; 4 Quality Assurance and Safety: Medicines, Department of Essential Medicines and Pharmaceutical Policies, World Health Organization, Geneva, Switzerland; 5 Office of the US Global AIDS Coordinator, US Department of State, Washington, D.C., United States of America; 6 WHO Collaborating Centre for International Drug Monitoring, Uppsala, Sweden; 7 Department of HIV/AIDS, World Health Organization, Geneva, Switzerland; 8 WHO Collaborating Centre for Advocacy and Training in Pharmacovigilance, University of Ghana Medical School, Accra, Ghana; 9 Ranbaxy Inc., Princeton, New Jersey, United States of America; 10 Management Sciences for Health, Strengthening Pharmaceutical Systems (SPS) Programs, Arlington, Virginia, United States of America; 11 Graduate Studies Program in Epidemiology, Federal University of Rio Grande do Sul, Hospital de Clinicas de Porto Alegre, Porto Alegre, Brazil, and Brazilian National Institute of Science and Technology for Health Technology Assessment (IATS CNPq), Porto Alegre, Brazil; 12 New York University School of Medicine, New York, New York, United States of America; 13 Forum for Collaborative HIV Research, Washington, D.C., United States of America

## Abstract

Jur Strobos and colleagues describe the deliberations of a recent multi-stakeholder meeting discussing the creation of a sustainable global pharmacovigilance system for antiretroviral drugs that would be applicable in resource limited settings.

**Summary Points**

- With increasing numbers of people worldwide on antiretroviral drugs, the need for improved and sustained global drug safety monitoring or pharmacovigilance is critical.
- Pharmacovigilance includes monitoring for substandard products, diversion, inappropriate use, and toxicity and is an essential component of safe and effective drug usage.
- The Forum for Collaborative HIV Research was asked to use its neutral setting for key stakeholders from the UN and government agencies, donors, industry, academia, multilateral organizations, and implementers to discuss the creation of a sustainable global pharmacovigilance system for ARVs.
- Important but contrasting priorities and values among stakeholders—all of whom are dedicated to establishing global pharmacovigilance—were identified as barriers to progress.
- Recognition, understanding, and respect for these contrasts is a pathway for increased collaboration and cooperation that will then lead to a sustainable system involving all stakeholders including industry and experienced regulatory agencies.

## Background

As of 2010, over 5 million people worldwide have access to antiretroviral drugs (ARVs) [1]. With increased access comes a greater need to monitor and promote the safety and effectiveness of these essential medicines. Few resource-limited countries have all the structures, systems, or resources necessary to support medicines safety activities [2],[3]. Diverse international efforts to provide optimal treatment could be compromised by the absence of robust strategies and frameworks for monitoring of inappropriate use, toxicity, drug-drug interactions, diversion, and substandard medicines [4]. While isolated episodes of substandard medication distribution have been identified and handled through the involvement of the World Health Organization (WHO), global pharmacovigilance is needed to fully understand the extent of the issue. Loss of confidence in the safety of ARVs could lead to poor adherence and the emergence of drug resistance, reduced demand for therapy, or inappropriate switching to more toxic or expensive medicines. A sustainable pharmacovigilance system can help achieve comprehensive, safe, and effective healthcare. Efforts to date have attempted to address the need for responsive drug safety monitoring systems for ARVs in resource-limited settings (RLS) but with varying success. The Forum for Collaborative HIV Research (Forum) was asked to use its neutral setting for key stakeholders from the United Nations and government agencies, donors, industry, academia, multilateral organizations, and implementers to discuss the creation of a sustainable global pharmacovigilance system for ARVs that would be applicable in RLS. A meeting was convened by the Forum on June 11, 2010, that included relevant stakeholders to discuss barriers to progress. Stakeholder participants other than the authors are identified in the Acknowledgments.

## The Issue: Contrasting Priorities, Values, and Ideas

The Forum meeting participants discussed their approaches to establishing an acceptable and sustainable global pharmacovigilance framework for ARVs and how these efforts might be harmonized. The discussions revealed varieties of methods, opinions, and practices among the stakeholders. Challenges in integrating these different approaches and sub-optimal communication among stakeholders may have impeded progress in the past. Each of the values and ideas represent important technical, cultural, and economic imperatives that require mutual understanding and respect. The meeting participants agreed that understanding and mutual respect for others' priorities and ideas are the predicate to effective communication, then follows collaboration, leading to resolution or compromise and eventual success. At the Forum meeting, some of these contrasting values were highlighted:

*1. National sovereignty versus regional and international collaboration*

Sovereignty, country ownership, and building national infrastructure are values that have governed international collaboration since the founding of the United Nations. The WHO, the President's Emergency Program for AIDS Relief (PEPFAR), the Global Fund to Fight AIDS, Tuberculosis and Malaria (Global Fund), and the Uppsala Monitoring Center/WHO Collaborating Centre for International Drug Monitoring (UMC) all value national sovereignty. Their remit has been to work with national systems wherever possible. They also recognize that the need for data sharing and resource efficiency in some cases is best done through collaboration among regional and international stakeholders who provide systems, expert personnel, and other resources.

*2. The value of pharmacovigilance versus the needs for care delivery*

Optimal use of all available resources to deliver safe and effective HIV treatment and prevention is a priority. Safe and effective use of medicines is only guaranteed when access goes hand-in-hand with pharmacovigilance. Many stakeholders perceive the cost of pharmacovigilance infrastructure as competing with distribution of scarce human and financial resources for direct care delivery. This perception may impede the devotion of time and resources to development of sustainable global pharmacovigilance.

*3. Research, pharmacovigilance, and programmatic funding*

Scarce funds are distributed through diverse channels whose distinctions may be artificial in the resource-limited world. Established health care systems have separate channels for funding for research, pharmacovigilance, and operations. Research monies are mainly derived from national scientific or medical agencies, industry research, and foundations. Pharmacovigilance is largely funded by the operational side of the pharmaceutical industry and by health ministries. Health care operations are funded by insurance companies, donors, and national health systems. The absence of global pharmacovigilance systems, the need to develop systems that may differ from well-established models, and the need to assess the feasibility of novel models offer opportunities for innovation. Barriers among these channels of funding in established systems are not applicable in RLS. Sustainable global pharmacovigilance must derive support from operational, research, *and* programmatic funds as programs, such as health care delivery and pharmacovigilance, are inextricably intertwined in this setting with the need for epidemiological and implementation research [5].

*4. Active versus spontaneous surveillance*

Active surveillance with intensive data collection is one proven method of systematically identifying and assessing medication use and patient outcomes. Spontaneous surveillance collects reporters' concerns about an event seen during treatment, assesses clinical causality, and provides timely information on low incidence adverse events that manifest upon exposure of large numbers of patients to drug products. Spontaneous reporting can be time consuming and adds to the workload of already overburdened healthcare professionals in RLS. In these instances, monitoring through a sentinel site might be a viable alternative. Active surveillance of cohorts [6] or through the use of registries (e.g., the established Antiretroviral Pregnancy Registry) [7] may be more expensive but provides a mechanism for assessing incidence and to conduct pharmacovigilance within special populations. While countries with established pharmacovigilance can employ both systems effectively, the best mix for RLS still requires careful planning.

*5. Confidentiality of safety data versus need for transparency and public access*

Regulatory agencies in Europe receive confidential safety data from pharmaceutical companies for registration purposes and afterwards in post-marketing surveillance. Confidentiality may be important to assure that adverse event relationships that are later understood to be associative rather than causative do not create undue public confusion or alarm. But, international data sharing is necessary to support global pharmacovigilance, particularly given international trade and traffic in pharmaceuticals.

*6. Industry support versus global enforcement of reliable reporting*

The pharmaceutical industry plays an active role in funding pharmacovigilance in many nations. In respect of national sovereignty and, possibly, to avoid industry conflicts of interest in reporting events that may undermine investor confidence, global pharmacovigilance systems have not drawn upon industry funding and collaboration. In countries with established drug regulation, the conflict is partly controlled by audit, laws, regulations, and enforcement—a system not necessarily available globally. A global system that enjoys reliable industry engagement must draw upon new or shared sources for funding of enforcement.

*7. Generic versus innovator antiretroviral manufacturers*

Generic companies provide a significant proportion of ARVs distributed in RLS. The lower pricing ensures greater access [8]. But, correspondingly, these companies may devote fewer resources for pharmacovigilance. Established systems rely upon innovator companies to support pharmacovigilance on market entry, but this may not work in RLS where innovator companies may not be marketing their compounds.

## Progress

There has been great progress in international identification of the *need* for collaboration and renewed vigor in pursuing a global pharmacovigilance system—an important outcome of the Forum meeting. This is reflected in recent and ongoing developments in intergovernmental assistance programs as well as initiation support from the WHO and allied international and national entities. The Forum's meeting recognized ongoing efforts for collaborative pharmacovigilance among less resource-rich nations. In November 2009, a WHO-Global Fund Pharmacovigilance Strategy was drafted that identified the elements and roadmap for a sustainable, global partnership for system-driven pharmacovigilance [9]. The concept paper together with the minimum pharmacovigilance requirements for countries in RLS were presented for consideration at a Pharmacovigilance Stakeholders Meeting in November 2010, in Accra, Ghana. Participants included many of those present at the Forum meeting and were invited to comment. Progress was made on encouraging regional systems and the inclusion of international resources [10],[11]. There was support for the continued development of UMC as a global resource for pharmacovigilance activities.

The US Agency for International Development (USAID)-funded Strengthening Pharmaceutical Systems (SPS) program implemented by Management Sciences for Health sponsored a conference in Nairobi, Kenya, entitled “National Pharmacovigilance Systems: Ensuring the Safe Use of Medicines,” on the implementation of pharmacovigilance systems from a country-centered perspective. The conferees discussed a framework for pharmacovigilance and the need for performance metrics—the Indicator-based Pharmacovigilance Assessment Tool (IPAT)—and experiences and best practices were shared by participants from 30 countries.

The first version of a Web-based “Pharmacovigilance Toolkit,” developed by the WHO Collaborating Centre for Advocacy and Training in pharmacovigilance (the WHO CC/UMC-Africa) along with SPS and other partners, was presented at the Stakeholders meeting in Accra [12].

More recently, WHO has initiated two major projects in pharmacovigilance with the support of the Bill & Melinda Gates Foundation (BMGF). A pilot sentinel cohort in Tanzania established with the collaboration of UMC and the national health ministry may be open to patient enrollment soon. Second, the US National Institute of Allergy and Infectious Diseases (NIAID) is working collaboratively with WHO to establish spontaneous adverse event reporting in their IeDEA patient cohorts in two countries with an aim of evaluating and improving an abbreviated reporting system developed by UMC (CEMFlow). The Global Fund has also supported a start-up pharmacovigilance program for ARVs in the Ukraine.

Monitoring Medicines, a project funded by the European Commission, brings together 11 partners, including WHO, the UMC, and the Copenhagen HIV Programme, to advance pharmacovigilance within and outside the European Union (EU) [13]. The 14 work packages within this project consider various issues such as tools to support public reporting of adverse drug reactions; an electronic platform that consolidates HIV ADR information from several sources (http://www.hivpv.org/); and an algorithm for the detection of substandard and counterfeit medicines from pharmacovigilance data.

Finally, the US National Institutes of Health, the Global Fund, UMC, and a consortium of North American universities led by the University of Indiana (funded by PEPFAR through USAID) have collaboratively initiated a pharmacovigilance program with the Kenyan national health ministry. This illustrates, as well, the growing number of academic institutions that are engaged in pharmacovigilance: advancing methods, creating an evidence-base for assessing and improving medicines safety, performing statistical and data analyses, and training practitioners.

## Much Remains to Be Done

More can be achieved. Principally, there is hope for more inclusion of non-national stakeholders and experienced regulators and, perhaps more importantly, for assurance of financial and institutional sustainability. Many stakeholders with substantial interests are still not routinely at the table. Most prominently, the pharmaceutical industry, both innovator and generic, are not included, although both sets of institutions have substantial investment in global infrastructure, personnel, data management, databases, and other resources that could be purposed, at least in part, to sustaining global pharmacovigilance systems in RLS. Well-established national regulatory authorities, such as the European Medicines Agency (EMA), the US Food and Drug Administration (FDA), and the WHO Prequalification Programme also have data, expertise, human resources, and technical capacity that could be used more systematically to support pharmacovigilance in RLS. The EU has provided research and development money for pharmacovigilance. This funding must be continued in future rounds of EU funding. More transparent and proactive mechanisms for drug evaluation at all national regulatory agencies may also enhance support for comprehensive and sustainable pharmacovigilance. Those mechanisms may also raise awareness of the importance of pharmacovigilance.

Current projects need sustainable, if not growing, support. BMGF research funding will largely end in 2012. Round 11 of the Global Fund is unlikely to result in initiation of new pharmacovigilance programs. PEPFAR currently supports the clinical care and treatment of over 3.2 million people and has funded the strengthening of supply chains and access to pharmaceuticals. PEPFAR also supports some WHO pharmacovigilance activities but is also exploring ways to collaboratively fund pharmacovigilance systems to further strengthen the quality of national HIV programs. National laws, remits of international agencies, and funding authorizations may need to specifically address the need for funding of sustainable global pharmacovigilance. Still, too little is understood about the value and need for global pharmacovigilance and more must be done among funding organizations, including national bodies in resource-rich nations with established systems. Better linkages between disease-driven national programs (e.g., HIV/AIDS) and national pharmacovigilance centers should be made. While BMGF and NIAID have provided research funding, barriers between the purpose of those funds and investigations into sustainable systems and funding for those systems remain. Decisions on funding must include participation and collaboration with academic researchers, industry, donors, and well-resourced existing pharmacovigilance systems. Pharmaceutical industry resources must be more effectively recruited and used in implementation, whether in terms of international and national mandates or in terms of in-kind technical support.

Stakeholder collaboration, communication, and joint activity are developing rapidly. Stakeholders must continue to work together and communicate. There must be a formalized process comparable to the Forum-initiated consortium to ensure ongoing communication among all stakeholders that includes established national regulators and the innovator and generic companies. A workable system will not be necessarily based on each resource-limited national entity or regional program developing a separate system—there are simply insufficient technical, management, and funding resources. Instead, the stakeholders must collaboratively insist on broader systems that provide local value. These systems should be highly valued and be supported by industry, governments and funders of HIV programs and national health systems, multilateral organizations, and other key stakeholders, and should be funded even during difficult economic times.

## Abbreviations

ARV: antiretroviral drug
EU: European Union
Global Fund: Global Fund to Fight AIDS, Tuberculosis and Malaria
PEPFAR: President's Emergency Program for AIDS Relief
RLS: resource-limited settings
SPS: Strengthening Pharmaceutical Systems
UMC: Uppsala Monitoring Center/WHO Collaborating Centre for International Drug Monitoring
WHO: World Health Organization
